# Supplementary material for: Synaptic mechanisms underlying modulation of locomotor-related motoneuron output by premotor cholinergic interneurons
Source: eLife. 2020 Feb 21;9:e54170. doi: 10.7554/eLife.54170 (PMC7062467; doi:10.7554/eLife.54170)
Supplement: Supplementary file 1. [file elife-54170-supp1.docx]

Table 1 - Table 1 – Intrinsic properties of lumbar motoneurons from *hM3Dq* and *Pitx2::Cre; hM3Dq* mice

|  |  | |
| --- | --- | --- |
|  | *hM3Dq* | *Pitx2::Cre; hM3Dq* |
| Resistance (MΩ) | 63±3 | 58±4 |
| Capacitance (pF) | 114±7 | 113±4 |
| Membrane potential (mV) | -60±2 | -62±2 |
| Rheobase (pA) | 263±43 | 353±43 |
| Depolarizing block (pA) | 666±44 | 817±84 |
| Maximum firing (Hz) | 26±2 | 27±2 |
|  | n=16 | n=23 |
|  |  |  |
|  |  |  |

Table 2 - Intrinsic properties of Pitx2^+^ interneurons from control, excitatory and inhibitory DREADD mice

|  | *Pitx2::Cre;tdTomato* | *Pitx2::Cre;tdTomato;hM4Di* | *Pitx2::Cre;tdTomato;hM3Dq* |
| --- | --- | --- | --- |
| Resistance (MΩ) | 336±48 | 307±30 | 320±16 |
| Capacitance (pF) | 28±2 | 35±3 | 26±4 |
| Membrane potential (mV) | -52±3 | -52±2 | -53±1 |
| Spontaneous firing (Hz) | 2.2±0.6 | 1.8±0.5 | 2.0±0.4 |
|  | n=11 | n=9 | n=9 |

Table 3 - Ventral root burst frequency and duration in the presence of NMDA, 5-HT and DA of *hM4Di* and *Pitx2::Cre;hM4Di* mice

|  | *hM4Di* | *Pitx2::Cre;hM4Di* |
| --- | --- | --- |
| Burst frequency (Hz) | 0.23±0.03 | 0.21±0.03 |
| Burst duration (ms) | 2049±507 | 2393±554 |
|  | n=14 | n=19 |
